# In vivo spatial coordination with synthetic paracrine signaling

**DOI:** 10.64898/2026.06.26.734902

**Published:** 2026-08-27

**Authors:** Kaiwen Luo, Yitong Ma, Hongyi R. Li, Hengyu Li, Mengziang Lei, Margaret B. Swift, Nathan F. Dalleska, Abdullah S. Farooq, Ernesto Criado-Hidalgo, Ann Liu, Mikhail G. Shapiro, Michael B. Elowitz

## Abstract

The immune system uses paracrine signaling to spatially confine potent responses such as inflammation. A bio-orthogonal synthetic paracrine system could enable engineering of analogous multicellular circuits in which different cell types coordinate their functions in a spatially organized fashion. Here, using the plant hormone auxin as a bio-orthogonal chemical signal, we introduce programmable paracrine circuits that distribute sensing and effector functions to different cell types to spatially restrict responses in mouse xenografts. Cells engineered to express auxin biosynthetic genes generated auxin-dense regions with tunable length scales in vivo. This localized signaling ability enabled design of a multicellular sentinel-effector system, in which THP-1 sentinel cells conditionally produce auxin in regions expressing the tumor-specific antigen EGFRvIII, and Jurkat effector cells respond by locally modulating the activity of a chimeric antigen receptor (CAR). This two-cell type system was able to achieve localized activation of engineered effector cells in vivo. These results establish a foundation for engineering multicellular therapeutic systems that focus responses in specific tissue contexts or disease sites.

## Full Text Availability

The license terms selected by the author(s) for this preprint version do not permit archiving in PMC. The full text is available from the preprint server.

