# Supplementary material for: In vivo spatial coordination with synthetic paracrine signaling
Source: bioRxiv. 2026 Jun 29:2026.06.26.734902. Preprint. [Version 1] doi: 10.64898/2026.06.26.734902 (PMC13345098; doi:10.64898/2026.06.26.734902)
Supplement: Supplement 1 [file NIHPP2026.06.26.734902v1-supplement-1.pdf]

bioRxiv preprint doi: https://doi.org/10.64898/2026.06.26.734902; this version posted June 29, 2026. The copyright holder for this preprint (which was not certified by peer review) is the author/funder, who has granted bioRxiv a license to display the preprint in perpetuity. It is made available under a CC-BY 4.0 International license.

Supplementary Information for:

In vivo spatial coordination with synthetic paracrine signaling

Kaiwen Luo[1,8], Yitong Ma[2,8], Hongyi R. Li[1], Hengyu Li[3], Margaret B. Swift[3], Nathan F. Dalleska[4], Abdullah S. Farooq[1], Ernesto Criado-Hidalgo[3], Ann Liu[1], Mikhail G. Shapiro[3,5,7]*, and Michael B. Elowitz[1,6,7,9]**

[1]Division of Biology and Bioengineering, California Institute of Technology
[2]Department of Bioengineering, Stanford University
[3]Division of Chemistry and Chemical Engineering, California Institute of Technology
[4]Division of Geological and Planetary Sciences, California Institute of Technology
[5]Andrew and Peggy Cherng Department of Medical Engineering, California Institute of Technology
[6]Division of Engineering and Applied Science, California Institute of Technology
[7]Howard Hughes Medical Institute, California Institute of Technology
[8]These authors contributed equally
[9]Lead contact
*Correspondence: mikhail@caltech.edu
**Correspondence: melowitz@caltech.edu

Figure S1 relates to the CHO sender/receiver module (**Fig. 2**).
Figure S2 describes THP-1 constitutive sender optimization, supporting **Figure 3**.
Figure S3 is related to **Figure 4**.
Figure S4 is relevant to **Figure 5**.

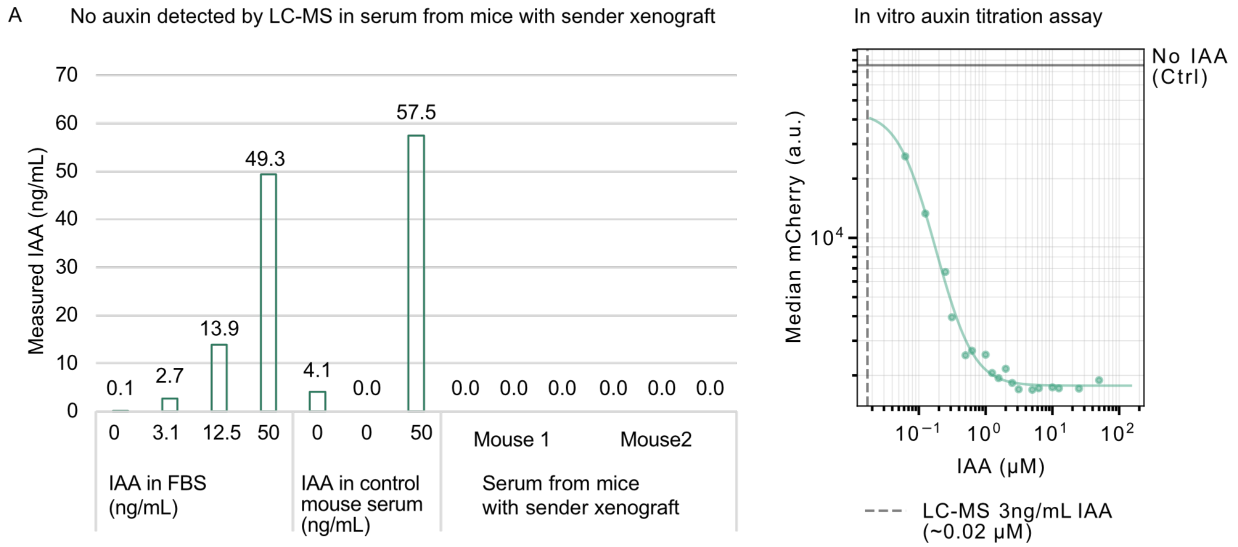

Figure S1: Auxin remains largely undetectable in serum. (**A**) (Left) Serum auxin was measured by liquid chromatography-mass spectrometry (LC-MS) from mice bearing xenografts containing 10% senders/90% receivers and was negligible. As controls, FBS and serum from receiver-only mice were spiked with defined IAA concentrations. Controls were N = 1 per matrix; CHO sender Mouse1 and Mouse2 were biological replicates, each measured in technical triplicates. An IAA spike of 3 ng/mL corresponds to ~0.02 μM, and is insufficient to elicit a strong receiver response (right). Note that in raw data, all samples had some peak area signal, but some were shown as 0 because the area values were at/below the background level, as determined by the calibration curve (**Supplementary Data**). Hill function was used to fit data points (**Methods**). See **Supplementary Data** for fitted parameters.

bioRxiv preprint doi: https://doi.org/10.64898/2026.06.26.734902; this version posted June 29, 2026. The copyright holder for this preprint (which was not certified by peer review) is the author/funder, who has granted bioRxiv a license to display the preprint in perpetuity. It is made available under a CC-BY 4.0 International license.

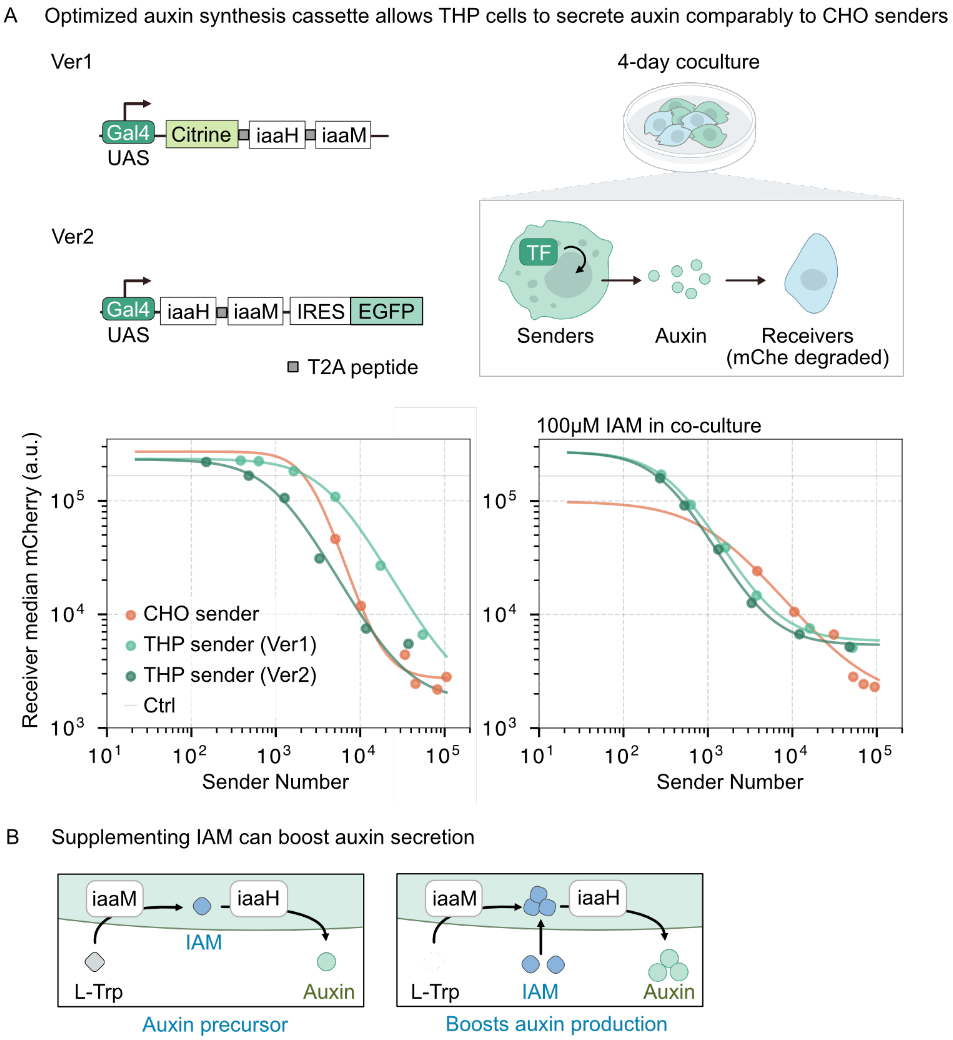

Figure S2: Optimized auxin production construct allows constitutive THP auxin senders to secrete auxin at levels comparable to CHO senders. (**A**) Different constitutive auxin senders (Ver1/Ver2) were co-cultured with CHO auxin receivers for 4 days. The cells then underwent flow cytometry to compare auxin secretion strength (receiver mCherry-AID degradation). IAM was applied in co-culture to investigate if the improved performance in the optimized construct is due to improved iaaM activity. Hill function was used to fit data points (**Methods**). See **Supplementary Data** for fitted parameters. N = 1 for each concentration group for the titration curves. (**B**) IAM, as the auxin precursor, is supplied to boost auxin production.

bioRxiv preprint doi: https://doi.org/10.64898/2026.06.26.734902; this version posted June 29, 2026. The copyright holder for this preprint (which was not certified by peer review) is the author/funder, who has granted bioRxiv a license to display the preprint in perpetuity. It is made available under a CC-BY 4.0 International license.

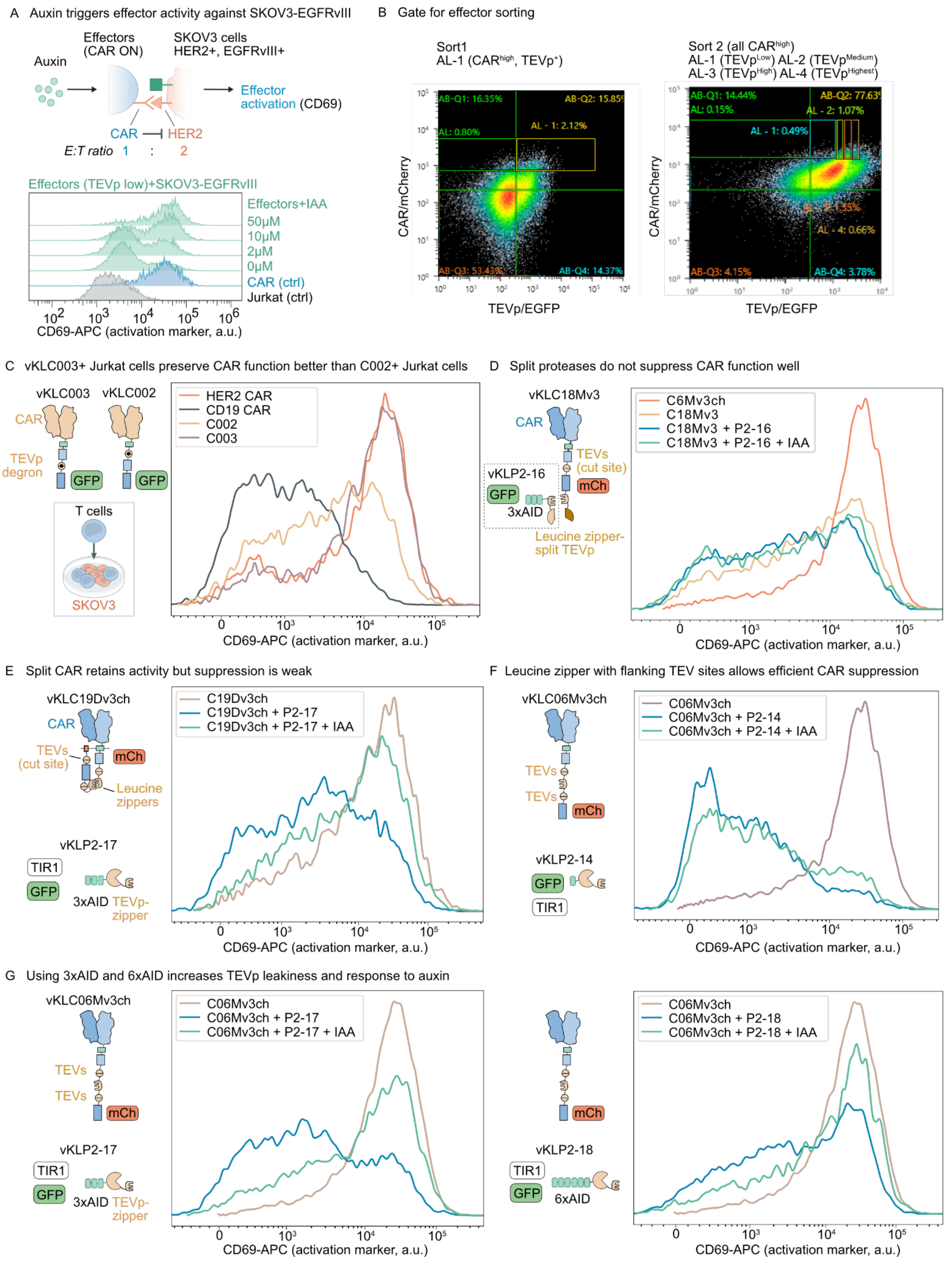

Figure S3: Effector design iteration, additional validation and expression optimization by sorting. (**A**) Same co-culture setup as in **Fig. 4D**, but using EGFRvIII+ SKOV3 cells to examine auxin-gated CAR activity in the presence of EGFRvIII. (**B**) Gate used for effector sorting. (**C-G**) Different CAR/TEVp designs leading towards the final design. Jurkat cells were transduced with corresponding lentiviruses encoding the constructs, co-cultured with SKOV3 cells in a 1:2-1:5 ratio. The cells were pre-treated with auxin for 24 h before another 24 h co-culture with SKOV3 cells. The cells were gated on CAR+ based on the corresponding construct-linked fluorescence marker (EGFP or mCherry). IAA, indole-3-acetic acid (auxin). N = 1 for each example.

bioRxiv preprint doi: https://doi.org/10.64898/2026.06.26.734902; this version posted June 29, 2026. The copyright holder for this preprint (which was not certified by peer review) is the author/funder, who has granted bioRxiv a license to display the preprint in perpetuity. It is made available under a CC-BY 4.0 International license.

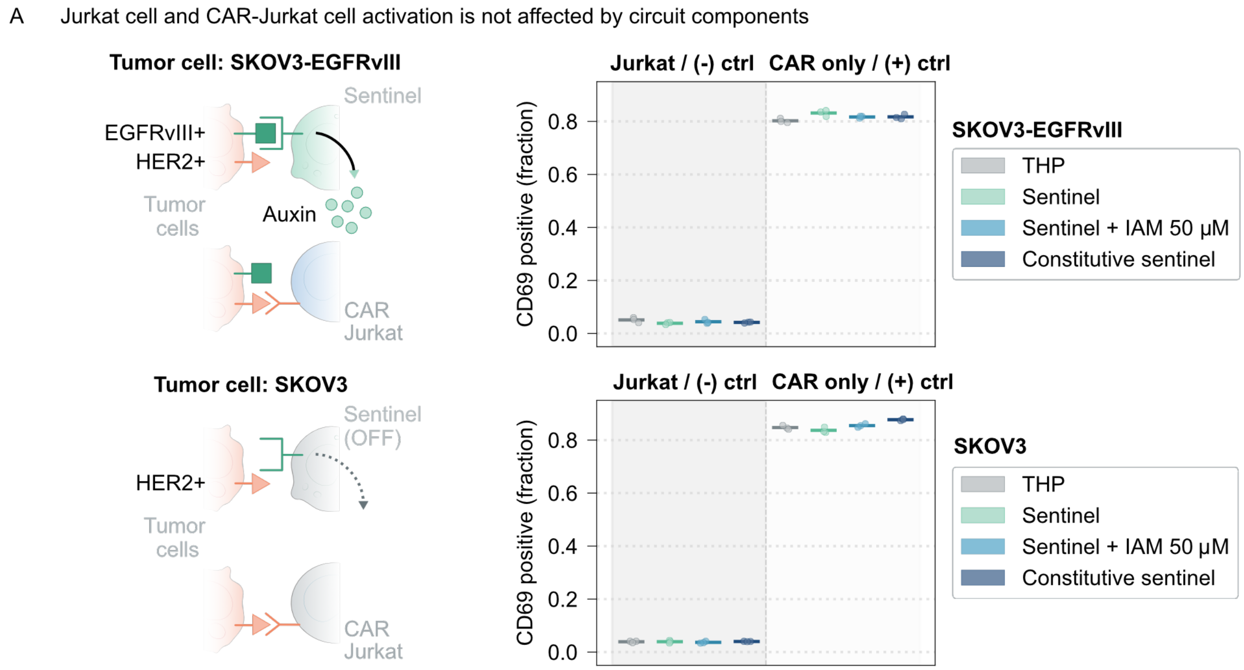

Figure S4: The activation of CAR-only Jurkat and wild-type Jurkat cells is independent of the presence of EGFRvIII or auxin production. (**A**) The co-culture and readout setup was the same as **Fig. 5A**, but with Jurkat effector cells replaced by CAR Jurkat cells without TEVp or wild-type Jurkat cells, as control groups. N = 3 for each condition.
